# Statistical Analysis of Biomarkers for Personalized Medicine

**DOI:** 10.1155/2013/467420

**Published:** 2013-11-06

**Authors:** Shinto Eguchi, Shigeyuki Matsui, Su-Yun Huang, Chuhsing Kate Hsiao

**Affiliations:** ^1^Department of Mathematical Analysis and Statistical Inference, Institute Statistical Mathematics, Tokyo 190-8562, Japan; ^2^Graduate School of Medicine, Nagoya University, Nagoya 466-8550, Japan; ^3^Institute of Statistical Science, Academia Sinica, Taipei 11529, Taiwan; ^4^Institute of Epidemiology and Preventive Medicine, National Taiwan University, Taipei 10617, Taiwan

Recently there has been enhanced and advanced biomedical technology such as high-throughput microarrays and molecular imaging to monitor SNPs, gene and protein expressions, and so forth, to provide exhaustive situations for individuals. In principle we could get much more information to know the biological and medical status from such data sets, which are viewed as biomarkers in a wide sense to help to do identification, association, and prediction studies for phenotypes such as cancer subtypes, prognosis, treatment responsiveness, and adverse reactions for personalized medicine. However, it is frequently difficult to extract only an informative part in the hull of data sets which include a garbage heap of uninformative observations. In particular, we cannot confirm statistical evidence because of the small sample size relative to the dimension of the data when we achieve the practical use of such statistical applications. 

For example, if we implement machine learning methods for the prediction of the treatment effects, then we typically face difficulties to confirm the reproducibility and robustness for the performance. In the meanwhile, effective study design is crucial for developing and validating biomarkers. In effect we present eight papers challenging this difficult situation of statistical analysis for biomarkers including statistical estimation, prediction, and testing hypothesis.

The paper, “*The number of candidate variants in exome sequencing for Mendelian Disease under no genetic heterogeneity,*” by J. Nishino and S. Mano studies the expected number of candidate single nucleotide variants (SNVs) in exome data for autosomal dominant or recessive Mendelian disorders so that the filtering method is useful with an assumption of “no genetic heterogeneity.”

The paper, “*Radial basis function-sparse partial least squares for application to brain imaging data,*” by H. Yoshida et al. proposes a novel statistical method, called radial basis function sparse partial least squares (RBF-sPLS), for investigating the relationship between clinical characteristics and brain morphology based on three-dimensional MRI data.

The paper, “*Power analysis of C-TDT for small sample size genome-wide association studies by the joint use of case-parent trios and pairs,*” by F. Rajabli et al. addresses family-based genetic association studies with missing genotype information for one of the parents. The paper provides a simulation study for the disequilibrium test statistic across different sample sizes for a family-based genome-wide association study. 

The paper, “*Genomic biomarkers for personalized medicine: development and validation in clinical studies*” by S. Matsui addresses the two critical statistical analyses of high-dimensional genomic data, gene screening and prediction, in the framework of development and validation of genomic biomarkers or signatures with biomarker-based clinical trial designs to assess clinical utility of a biomarker or a new treatment with a companion biomarker.

The paper, “*Multiple suboptimal solutions for prediction rules in gene expression data,*” by O. Komori et al. reanalyzes the gene expression data for pattern recognition of phenotypes to extract informative features embedded in the data. The paper concludes that it is not possible to extract only informative genes with high performance in the all observed genes and points out that the mutual coherence among genes is too high to find the solution of informative genes.

The paper, “*An empirical Bayes optimal discovery procedure based on semiparametric hierarchical mixture models,*” by H. Noma and S. Matsui proposes an empirical Bayes optimal discovery procedure with the smoothing-by-roughening approach in semiparametric hierarchical mixture models. This approach provides analytically a tractable posterior distribution with the flexible prior section in genome-wide studies.

The paper, “*Cancer outlier analysis based on mixture modeling of gene expression data,*” by K. Mori et al. proposes a gene-based statistic for gene selection on the basis of a posterior probability of cancer outlier for each cancer sample with an application to real data from hematologic malignancies.

The paper, “*A robust rerank approach for feature selection and its application to pooling-based GWA studies,*” by J. Liu et al. proposes a method based on the concept of rank-over-variable which is shown to be insensitive to the selection of tuning parameters compared with *t*-statistics, AUC statistics, and SAM with simulation studies and real data analysis of pooling-based genome-wide association (GWA) study to demonstrate the usefulness of their method.

*Shinto Eguchi* *Shigeyuki Matsui* *Su-Yun Huang* *Chuhsing Kate Hsiao*

